# Early Oral Antibiotic Switch in *Staphylococcus aureus* Bacteraemia: The *Staphylococcus aureus* Network Adaptive Platform (SNAP) Trial Early Oral Switch Protocol

**DOI:** 10.1093/cid/ciad666

**Published:** 2023-10-31

**Authors:** Dana de Kretser, Jocelyn Mora, Max Bloomfield, Anita Campbell, Matthew P Cheng, Stephen Guy, Marjolein Hensgens, Shirin Kalimuddin, Todd C Lee, Amy Legg, Robert K Mahar, Michael Marks, Julie Marsh, Anna McGlothin, Susan C Morpeth, Archana Sud, Jaap Ten Oever, Dafna Yahav, Marc Bonten, Asha C Bowen, Nick Daneman, Sebastiaan J van Hal, George S Heriot, Roger J Lewis, David C Lye, Zoe McQuilten, David L Paterson, J Owen Robinson, Jason A Roberts, Matthew Scarborough, Steve A Webb, Lynda Whiteway, Steven Y C Tong, Joshua S Davis, Genevieve Walls, Anna L Goodman, the SNAP Early Oral Switch Domain-Specific Working Group and SNAP Global Trial Steering Committee, J Marsh, S Y C Tong, J S Davis, A L Goodman, G Walls, S C Morpeth, M Hensgens, J Mora, D Yahav, A McGlothlin, M P Cheng, for the SNAP Trial Group, Nick Anagnostou, Sophia Acrhuleta, Eugene Athan, Lauren Barina, Emma Best, Katie Brett, Hannah Burden, Peter Daley, Jane Davies, P Partha De, Yael Dishon-Benattar, Katie Flanagan, Jennifer Grant, Dan Gregson, Kate Grimwade, James Hatcher, Andrew Henderson, Dina Jankovic, Jennie Johnstone, I Russel Lee, Ka Lip Chew, Martin Llewelyn, Anne-Grete Martson, Colin McArthur, Diana McNeil, Sarah Metcalf, Clare Nourse, Matthew O’Sullivan, Lina Petrella, Sarah Pett, Benjamin A Rogers, James Sim, Marta O Soares, Neil Stone, Robert Tilley, Rebecca Turner, Viliame Tutone, Jonathan Underwood, Lesley Voss, Rachel H Webb, Heather Wilson, Terence Wuerz

**Affiliations:** Medical Research Council Clinical Trials Unit, University College London, London, United Kingdom; Department of Infectious Diseases University of Melbourne, Peter Doherty Institute for Infection and Immunity, Melbourne, Australia; Department of Infection Services, Wellington Regional Hospital, New Zealand; Telethon Kids Institute, Wesfarmers Center of Infectious Diseases and Vaccines, The University of Western Australia, Perth, Australia; Divisions of Infectious Diseases and Medical Microbiology, McGill University Health Center, Montreal, Canada; Department of Infectious Diseases, Eastern Health, Box Hill, Australia; Monash University (including Australian and New Zealand Intensive Care Research Centre), Clayton, Australia; UMC Utrecht, Utrecht University, Utrecht, The Netherlands; Julius Center for Health Sciences and Primary Care, University Medical Centre Utrecht, Utrecht, The Netherlands; Department of Infectious Diseases, Singapore General Hospital, Singapore, Singapore; Program in Emerging Infectious Diseases, Duke-NUS Medical School, Singapore, Singapore; Clinical Practice Assessment Unit and Division of Infectious Diseases, McGill University, Montreal, Canada; Menzies School of Health Research, Charles Darwin University, Darwin, Northern Territory, Australia; Herston Infectious Diseases Institute, Herston, Brisbane, Australia; Centre for Epidemiology and Biostatistics, Melbourne School of Population and Global Health, University of Melbourne, Parkville, Australia; Clinical Epidemiology and Biostatistics Unit, Murdoch Children's Research Institute, Parkville, Australia; Department of Clinical Research, London School of Hygiene & Tropical Medicine, London, United Kingdom; Hospital for Tropical Diseases, University College London Hospital, London; Division of Infection and Immunity, University College London, London; Telethon Kids Institute &/Department of Infectious Diseases &/Wesfarmers Centre for Vaccines and Infectious Diseases, Perth Children's Hospital, Perth, Australia; Berry Consultants, LLC, Austin, Texas, USA; Department of Infectious Diseases, Middlemore Hospital, Auckland, New Zealand; Department of Infectious Diseases, University of Sydney, Nepean Hospital, Kingswood, New South Wales, Australia; Department of Internal Medicine and Radboud Centre for Infectious Diseases, Radboud University Medical Center Nijmegen, The Netherlands; Infectious Diseases Unit, Sheba Medical Center, Ramat-Gan, Israel; UMC Utrecht, Utrecht University, Utrecht, The Netherlands; Telethon Kids Institute &/Department of Infectious Diseases &/Wesfarmers Centre for Vaccines and Infectious Diseases, Perth Children's Hospital, Perth, Australia; Division of Infectious Diseases, Department of Medicine, Sunnybrook Health Sciences Centre, University of Toronto, Toronto, Canada; Department of Microbiology and Infectious Diseases, Royal Prince Alfred Hospital, Sydney, Australia; School of Medicine, University of Sydney, Sydney, Australia; Department of Infectious Diseases University of Melbourne, Peter Doherty Institute for Infection and Immunity, Melbourne, Australia; Berry Consultants, LLC, Austin, Texas, USA; National Center for Infectious Diseases, Singapore; Department of Infectious Diseases, Tan Tock Seng Hospital, Singapore; Yong Loo Lin School of Medicine, Singapore; Lee Kong Chian School of Medicine, Singapore; Monash University (including Australian and New Zealand Intensive Care Research Centre), Clayton, Australia; Department of Haematology, Monash Health, Melbourne, Australia; University of Queensland Centre for Clinical Research, Royal Brisbane and Women's Hospital Campus, Brisbane, Australia; Department of Infectious Diseases, Royal Perth Hospital, Perth, Australia; Department of Infectious Diseases, Fiona Stanley Hospital, Murdoch, Australia; PathWest Laboratory Medicine, Perth, Australia; College of Science, Health, Engineering and Education, Murdoch University, Murdoch, Australia; Herston Infectious Diseases Institute, Herston, Brisbane, Australia; University of Queensland Centre for Clinical Research, Royal Brisbane and Women's Hospital Campus, Brisbane, Australia; Metro North Health, Brisbane, Australia; Department of Pharmacy and Intensive Care Medicine, Royal Brisbane and Women's Hospital, Brisbane, Australia; Division of Anesthesiology Critical Care Emergency and Pain Medicine, Nîmes University Hospital, University of Montpellier, Nîmes, France; Department of Infectious Diseases, Oxford University Hospitals NHS Trust, Oxford, United Kingdom; Department of Infectious Diseases, Oxford University Hospitals NHS Trust, Oxford, United Kingdom; Department of Infectious Diseases University of Melbourne, Peter Doherty Institute for Infection and Immunity, Melbourne, Australia; Victorian Infectious Diseases Service, The Royal Melbourne Hospital, at the Peter Doherty Institute for Infection and Immunity, Melbourne, Australia; School of Medicine and Public Health and Hunter Medical Research Institute, University of Newcastle, Newcastle, Australia; Department of Infectious Diseases, Middlemore Hospital, Auckland, New Zealand; Medical Research Council Clinical Trials Unit, University College London, London, United Kingdom; Department of Infectious Diseases, Oxford University Hospitals NHS Trust, Oxford, United Kingdom; Centre for Clinical Infection and Diagnostics Research, Guy's and St Thomas' Foundation NHS Trust, King's College, London, United Kingdom

**Keywords:** *Staphylococcus aureus* (or *S. aureus*), bacteremia (or bacteraemia or bloodstream infection), adaptive platform clinical trial, oral antibiotic, intravenous antibiotic

## Abstract

**Background:**

*Staphylococcus aureus* bloodstream infection (bacteremia) is traditionally treated with at least 2 weeks of intravenous (IV) antibiotics in adults, 3–7 days in children, and often longer for those with complicated disease. The current practice of treating *S. aureus* bacteremia (SAB) with prolonged IV antibiotics (rather than oral antibiotics) is based on historical observational research and expert opinion. Prolonged IV antibiotic therapy has significant disadvantages for patients and healthcare systems, and there is growing interest in whether a switch to oral antibiotics following an initial period of IV therapy is a safe alternative for clinically stable patients.

**Protocol:**

The early oral switch (EOS) domain of the *S. aureus* Network Adaptive Platform (SNAP) trial will assess early switch to oral antibiotics compared with continued IV treatment in clinically stable patients with SAB. The primary endpoint is 90-day all-cause mortality. Hospitalised SAB patients are assessed at platform day 7 ±2 (uncomplicated SAB) and day 14 ±2 (complicated SAB) to determine their eligibility for randomization to EOS (intervention) or continued IV treatment (current standard of care).

**Discussion:**

Recruitment is occurring in the EOS domain of the SNAP trial. As of August 2023, 21% of all SNAP participants had been randomized to the EOS domain, a total of 264 participants across 77 centers, with an aim to recruit at least 1000 participants. We describe challenges and facilitators to enrolment in this domain to aid those planning similar trials.

## Teaser

*Staphylococcus aureus* bloodstream infection is treated with at least 2 weeks intravenous antibiotics in adults and a minimum 3–7 days in children. We aim to determine if an earlier switch to oral antibiotic therapy is safe for stable patients.

*Staphylococcus aureus* bacteraemia (SAB) is the leading cause of mortality due to bloodstream infections, [1]. Adult patients with SAB typically receive a minimum of 2 weeks of intravenous (IV) antibiotics. Patients with more complex infections may receive weeks of IV therapy [2, 3]. These durations are based on observational research and expert opinion [4]. The duration of IV treatment is shorter in children (median 11 days [5]), with oral switch occurring as early as day 3 of treatment in 1 small randomized clinical trial (RCT) [6].

IV administration is the most effective way to rapidly achieve therapeutic antibiotic concentrations in unwell patients with severe infections. Once the patient has stabilized, early transition to oral antibiotics is increasingly recognized as part of good antimicrobial stewardship (AMS) practice [3]. IV therapy requires substantial resources, is inconvenient and less acceptable to patients, and has risks such as venous catheter complications. Several antibiotics have excellent oral bioavailability, allowing achievement of plasma and tissue concentrations comparable to those achieved with IV administration. For antibiotics not traditionally considered highly orally bioavailable, dosing to optimize pharmacodynamic characteristics may enable acceptable antibiotic concentrations at the site of infection [7].

Two large RCTs of partial oral therapy—the POET endocarditis treatment study [8], which included *S. aureus* endocarditis, though was not powered to analyze this patient sub-group—and the SABATO study of uncomplicated SAB [9]—showed that partial oral therapy was non-inferior to extended IV treatment for these indications. Smaller RCTs including patients with *S. aureus* endocarditis [10] and SAB related to various sources [11], and several observational studies of complicated and uncomplicated SAB treatment have also demonstrated non-inferiority of early oral switch (EOS) [12–16] (Tables 1 and 2 contain a summary of literature on EOS in SAB). Of the oral antibiotics potentially available to treat SAB, the best evidence for clinical non-inferiority compared with IV treatment is for oral linezolid [20, 21]. Successful outcomes with an oral fluoroquinolone-rifampicin combination were reported in 2 studies of methicillin-susceptible *S. aureus* bacteremia (MSSA-B) [10, 11], and the RODEO-1 RCT will test this combination for treatment of endocarditis, including *S. aureus* endocarditis [22]. Other retrospective observational studies of partial oral treatment in SAB have included patients treated with oral trimethoprim-sulfamethoxazole [13, 14], clindamycin [16], and beta-lactams [8, 12] and have demonstrated the safety and efficacy of this approach for complicated and uncomplicated SAB.

**Table 1.** Published Studies Describing Oral Antibiotic Therapy for Uncomplicated or “Low-risk” *S. aureus* Bacteremia

Title (Author, Year, Title)	Study Design	Number and Category of Participants	Study Intervention	Study Outcome	Limitations
Willekens et al 2019, EOS to linezolid for low-risk patients with *Staphylococcus aureus* bloodstream infections. [15]	Prospective, single-center cohort study, non-randomized	152 low-risk SAB patients (45 EOS, 90 IV) 11% MRSA	EOS to oral linezolid 600 mg BD on day 3–9 of treatment or continued IV treatment	No significant difference in 90 d relapse or 30 d all-cause mortality between groups. Less time in hospital was required for the EOS group (8 d vs 19 d) EOS using linezolid gave similar clinical outcomes to continued IV treatment, and reduced hospital stay. Trend to improved outcomes (2% 30-day mortality vs 13%)	A relatively small sample size limited the power of the study
Thorlacius-Ussing et al 2019, Efficacy of 7 and 14 days of antibiotic treatment in uncomplicated *Staphylococcus aureus* bacteremia (SAB7). [17]	Randomised open label multicentre trial	Currently enrolling	RCT evaluating efficacy of 7 versus 14 d of antibiotic treatment for uncomplicated SAB. Antibiotic treatment and dosage are as per local or national guidelines.	No current update available.	…
Bupha-Intr et al 2020, Efficacy of EOS with beta-lactams for low-risk *Staphylococcus aureus* bacteraemia. [12]	Retrospective, single-centre, cohort study, non-randomised	100 low-risk SAB participants (84 EOS, 16 IV) 5% MRSA	EOS after median 5 d of IV and compared to patients who continued IV treatment. 86% of these received oral beta-lactams eg 1 g flucloxacillin TDS	Only 1 EOS incorrectly triaged patient relapsed in 90 d. No deaths attributable to SAB	These 100 were of 469 total patients. ‘Low-risk” included those with fever >48 h. EOS was standard of care, so the 16 IV participants were exceptions eg due to inability to absorb the antibiotic. Low MRSA (5%), a risk-factor for poor outcomes, were included in the cohort. Almost all participants suffered from line infections. Limited meaningful statistical comparisons due to the reduced size of the IV therapy group. No comment on side effects of either regimen
Kaasch et al 2023, EOS therapy in low-risk *Staphylococcus aureus* bloodstream infection (SABATO). [9]	Prospective, multi-centre, randomised, open label	213 low-risk SAB participants (108 EOS, 103 IV) 5% MRSA	EOS at Day 5–7 of treatment or standard continued IV treatment. Oral therapy TMP-SMX (160/800 mg BD), clindamycin (600 mg TDS) or (for MRSA) linezolid (600 mg BD). IV therapy for MSSA (flu)cloxacillin (2 g QDS) or cefazolin (2 g TDS), for MRSA vancomycin (1 g BD) or daptomycin (6–10 mg/kg OD).	No significant difference in 90-day SAB complications (primary outcome, 4% vs 5% of study population of 165) or death attributable to SAB (secondary outcome, 1% vs 0%) to oral versus IV respectively. Significantly shorter hospital stays were attributable to the EOS group (11 d vs 15 d).	Slow enrolment led to early termination and protocol changes during the trial- though the broadening to include a wider group of those at low-risk of SAB may have made it more applicable to the wider population.
Diego-Yague et al 2023, Sequential oral antibiotic in uncomplicated *Staphylococcus aureus* bacteremia: a propensity-matched cohort analysis. [18]	Retrospective, single-centre, cohort study, non-randomised	230 uncomplicated participants (112 EOS, 118 IV)	EOS after an median of 7 d. Most commonly co-amoxiclav alone (48%) but also cefadroxil, cefuroxime or cephalexin (all at 1 g TDS) (48%), ciprofloxacin alone (12%) and linezolid (9%) were the most commonly prescribed (other doses not stated).	Significant difference in composite 90-day all-cause mortality and 90-day microbiological failure (primary outcome, 10.7% (11/112) in OST versus 30.5% (36/118) in IVT (*P* < .001) but this significance was removed once propensity-matched cohort where the primary outcome occurred in 21% (32/154). No higher risk of microbiological relapse, readmission, or mortality.	The IV and EOS groups were propensity-matched, however not completely comparable, as mortality in the EOS cohort was lower than in the IV group. The relatively small sample size and observational nature of the study limits the strength of the conclusions.

Abbreviations: BD, administered twice a day; EOS, early oral switch; IV, intravenous; IVT, IV antibiotic therapy; MRSA, methicillin-resistant *Staphylococcus aureus*; MSSA, methicillin- susceptible *Staphylococcus Aureus*; OD, administered once a day; OST, oral sequential antibiotic therapy; QDS, administred four times a day; RCT, randomized clinical trial; SAB, *S. aureus* bacteraemia; SOC, standard of care; TDS, administered three times a day; TMP-SMX, trimethoprim-sulfamethoxazole.

**Table 2.** Published Studies Describing Oral Antibiotic Therapy for Complicated or “High-risk” *S. aureus* Bacteremia

Title (Author, Year, Reference)	Study Design	Number and Category of Participants	Study Intervention	Study Outcome	Limitations
Heldman et al. 1996, Oral antibiotic treatment of right-sided staphylococcal endocarditis in infection drug users: prospective randomized comparison with parenteral therapy [10]	A single centre (2 affiliated hospitals) prospective randomised trial.	Of note 61/89 recruited and tested had HIV. Of the total- 87 *S. aureus*, 5 MRSA, 6 Coagulase negative *Staphylococcus* (CoNS)	Prospective randomised trial of ciprofloxacin 750 mg BD + rifampicin 300 mg BD for right sided staphylococcal IE in PWID	Similar microbiological and clinical cure rates were noted compared with standard continued IV therapy	The sample size was too small, and confidence intervals too wide for statistical significance due to many patients not completing the protocol. The 2 deaths occurred in those not included, as they did not have right-sided endocarditis- both of whom switched very early at 24 h, and both died within 72 h. The doses used were much lower than those used in similar trials. Resistance to ciprofloxacin is a concern and may not have been adequately observed due to the low numbers of MRSA.
Schrenzel et al. 2004, A randomized clinical trial to compare fleroxacin + rifampicin with flucloxacillin or vancomycin for the treatment of staphylococcal infection. [11]	Multicentre, randomized trial	Deep-seated bacteraemic staphylococcal infections (mostly MSSA). 130 randomised, 127 treated. Not all SAB- CoNS included	Oral fleroxacin 400 mg OD + rifampicin 600 mg OD versus IV treatment for SAB	Similar cure rates, and microbiological/clinical failures were noted in both groups. Less time in hospital was required for the EOS group (12 d vs 23 d)	Fleroxacin is not a widely used drug, as not available in many countries. Extrapolation to newer fluoroquinolones should be valid but is uncertain.
Jorgensen et al. 2019, Sequential intravenous-to oral outpatient antibiotic therapy for MRSA bacteraemia: one step closer. [14]	Retrospective, single-centre study	492 complicated and uncomplicated MRSA-bacteraemia (MRSA-B) patients.	70 participants were switched to oral on discharge after median 8 d of IV treatment. 50% received PO linezolid and 34% PO TMP-SMX (doses not stated)	90-day failure rate non-significantly less in EOS group with significantly lower hospital readmission risk. Selected MRSA-B patients may have at least equivalent clinical outcomes with oral antibiotics versus OPAT	These patients were not part of a closed healthcare setting, limiting the ability to capture data or assess complications and readmissions at other institutions.
Iversen et al 2019, POET: Partial oral versus intravenous treatment of endocarditis. [8]	Randomised controlled trial	87 patients with *S. aureus* IE, and no MRSA	EOS versus continued IV treatment for IE: 54% switched to oral therapy after median 17 d Dicloxacillin/Amoxicillin (1 g QDS) + rifampicin (600 mg BD) was the most frequently used oral regime for *S. aureus* infection. IV therapy used a combination amoxicillin/dicloxacillin with another agent.	No difference in the composite primary outcome of all-cause mortality, unplanned cardiac surgery, embolic events, or relapse between the EOS and continued IV groups but not sub analysed for this.	We need to be careful of extrapolating this data, as this was not a planned sub analysis at start of trial.
Pérez-Rodríguez et al 2021, The benefits and safety of oral sequential antibiotic therapy in non-complicated and complicated *Staphylococcus aureus* bacteraemia. [13]	Retrospective, single-center	201 complicated and uncomplicated SAB patients (17% MRSA). Infective endocarditis (IE_) and endovascular infections were excluded.	62% switched to oral after median 13 d of IV; 66% of whom received TMP-SMX 160/800 mg BD. Others received linezolid (600 mg every 12 h), or levofloxacin (500 mg every 24 h)	No difference in cure rates, recurrence, or mortality were noted between groups. Shorter hospital stays were required for EOS group	Patients who died during their antibiotic therapy were excluded, possibly resulting in a population with less severe infection. Treatment duration was not factored into analysis, and it was noted that the EOS group remained on antibiotics for a greater period of time.
Kouijzer et al 2021, Intravenous to oral switch in complicated *Staphylococcus aureus* bacteraemia without endovascular infection: a retrospective single-centre cohort study. [16]	Retrospective single-center	106 patients with complicated SAB (96% MSSA), excluding IE/endovascular infection.	61% switched to oral antibiotics after a median 16 d IV; 88.5% of whom received PO clindamycin 600 mg TDS	No significant difference in 3-month mortality EOS versus IV group. No relapses observed among either group; Hospital stay reduced significantly by 12 d in EOS group.	Retrospective study design with a relatively small sample size introduces the inherent risk of confounding bias. Not all countries permit oral dosing of 600 mg clindamycin in each dose. Most applicable to isolates susceptible to clindamycin.
Wildenthal et al 2022, Outcomes of partial oral antibiotic treatment for complicated *Staphylococcus aureus* bacteremia in people who inject drugs. [19]	Retrospective, single-center, cohort analysis	238 patients with complicated SAB (infective endocarditis, septic arthritis, epidural abscess, and/or vertebral osteomyelitis) and a history of active or recent injection drug use.	69 participants were transitioned to partial oral antibiotic therapy after a median 18 d of IV antibiotics. Doxycycline 100 mg BD was used most frequently, followed by TMP-SMX 160/800 mg BD and Linezolid 600 mg BD; mostly as single-agent therapy.	SOC IV antibiotics and partial oral showed similar levels in microbiological failures. Patients who received at least 10 d of IV antibiotics prior to transition did not have significantly different outcomes. No difference between MRSA and MSSA, however, not powered for this analysis.	Date of discharge was used as the starting point for the follow up period which may have introduced immortal time bias. In addition, those that died before discharge were excluded, potentially biasing towards the IV antibiotic cohort.

Abbreviations: BD, administered twice a day; CoNS, Coagulase negative *Staphylococci*; EOS, early oral switch; HIV, human immunodeficiency virus; IE, infective endocarditis; IV, intravenous; MRSA, methicillin-resistant *Staphylococcus aureus*; MRSA-B, MRSA bacteraemia; MSSA, methicillin-susceptible *Staphylococcus aureus*; OD, administered once a day; OR, odds ratio; QDS, administred four times a day; SAB, *S. aureus* bacteraemia; SAB-CoNS, *S. aureus* bacteraemia coagulase negative *Staphylococcus*; SOC, standard of care; TDS, administered three times a day; TMP-SMX, trimethoprim-sulfamethoxazole.

In general, the existing studies have not been sufficiently powered to enable us to draw firm conclusions about the non-inferiority of EOS for outcomes such as mortality and treatment failure. To allow clinicians to confidently adopt the EOS paradigm for SAB treatment, there is a need to demonstrate the efficacy and safety of this strategy through a large, well-designed, international RCT such as SNAP.

SNAP consists of a single core protocol that governs overall trial conduct and separate appendixes allowing multiple domains to be embedded within it. This design enables the assessment of multiple clinical questions simultaneously. The estimated target sample size is 7000 participants [23]. It is a whole-of-life trial, enrolling neonates through to nonagenarians. This pragmatic trial design also includes groups traditionally excluded from trials, such as pregnant women and people who inject drugs. The EOS domain aims to determine whether EOS is non-inferior to continued IV treatment with respect to 90-day mortality, for patients with both uncomplicated and complicated bacteremia. Adverse events (including venous catheter-related adverse events) and patient-centered outcomes such as hospital length of stay will be collected. By virtue of its unprecedented sample size, SNAP should also be able to answer questions around timing of EOS and the selection of appropriate EOS candidates.

## METHODS: PARTICIPANTS, INTERVENTIONS, AND ENDPOINTS

The EOS domain-specific appendix was designed following the SPIRIT guidelines for trial protocols. The SNAP core protocol has been published [23], and the trial is registered on clinicaltrials.gov (NCT05137119). Herein we detail the protocol and rationale for the EOS domain but also refer the reader to the EOS protocol on the SNAP trial website.

### Participants

Participants already enrolled in SNAP (entry within 72 hours of collection of the first positive *S. aureus* blood culture) will be assessed against the eligibility criteria for the EOS domain, as shown in Table 3. Eligibility will be assessed at 2 time points, with differing criteria at each point: platform day 7 (±2) (D7) and platform day 14 (±2) (D14). The treating clinician and the investigator must be satisfied that the patient is clinically stable at either time point. Participants judged eligible at D7 are those with uncomplicated SAB; those judged eligible at D14 have complicated SAB (including endocarditis or osteomyelitis). For children, the EOS domain differs slightly in that it allows the inclusion of uncomplicated native osteoarticular infections in the D7 (or uncomplicated) group.

**Table 3.** EOS Domain Inclusion and Exclusion Criteria

Inclusion Criteria	Exclusion Criteria
Platform D7 (± 2 d)	
Clearance of SAB by platform Day 2: blood cultures negative for *S. aureus* from platform Day 2 onward AND no known subsequent positive blood cultures	Adherence to oral agents unlikely (as judged by site PI in consultation with the treating team)
Afebrile (<37.8°C) for the past 72 h (at time of judging eligibility). If there has been no documented evidence of fever, the site may consider that this inclusion criterion has been met	Unreliable gastrointestinal absorption (eg, vomiting, diarrhoea, nil by mouth, anatomical reasons)
*Adult:* Primary focus is either line related (either central or peripheral IV cannula) or skin and soft tissue, AND source control achieved (for “line-related” this means line removed; for “skin and soft tissue” means site PI considers source control to have been achieved and any abscess more than 2 cm diameter has been drained)	There are no appropriate oral antibiotics due to contraindications, drug interactions, drug availability, or antibiotic resistance
*Paediatrics:* Primary focus is either line related (either central or peripheral IV cannula), skin and soft tissue, or uncomplicated bone and joint infection AND source control achieved (for “line-related” this means line removed; for “skin and soft tissue” means site PI considers source control to have been achieved and any abscess more than 2 cm diameter has been drained, and for uncomplicated, native bone and joint infection either surgical drainage has occurred or the clinician deems this is not necessary)	Ongoing IV therapy unsuitable eg, no IV access
No evidence of metastatic foci (on clinical or radiological examination, but radiological imaging is not required to exclude metastatic foci if not clinically indicated)	Clinician deems not appropriate for EOS
…	Patient no longer willing to participate in domain
…	Clinical team deems that sufficient duration of antibiotic therapy has already been provided
…	Presence of prosthetic cardiac valve, pacemaker or other intracardiac implant
…	Known presence of intravascular clot, graft, or other intravascular prosthetic material
…	Intravascular/intracardiac infections
…	Presence of other intracardiac abnormalities felt to put patient at increased risk of endocarditis
Platform D14 (+/− 2 d)	
Clearance of SAB by platform Day 5: blood cultures negative for *S. aureus* from platform Day 5 (+/−1 d) AND no known subsequent positive blood cultures. If the most recent blood culture from Day 2–4 is negative for *S. aureus*, blood cultures do not need to be repeated on Day 5 to fulfil eligibility criteria (Day 5 blood cultures will be assumed to be negative in this situation)	Adherence to oral agents unlikely (as judged by site PI in consultation with the treating team)
Afebrile (<37.8°C) for the past 72 h (at time of judging eligibility). If there has been no documented evidence of fever, the site may consider that this inclusion criterion has been met.	Unreliable gastrointestinal absorption (eg, vomiting, diarrhoea, nil by mouth, anatomical reasons)
Site PI has determined that source control is adequate. This could include patients for whom the aim of treatment is long-term suppression rather than cure, for example, infected pacemaker wire or prosthetic joint where removal of wire or prosthesis is not possible (ie source control is appropriate for the treatment aim).	There are no appropriate oral antibiotics due to contraindications, drug interactions, drug availability, or antibiotic resistance
…	Ongoing IV therapy unsuitable, eg, no IV access
…	Clinician deems not appropriate for EOS (provide reason)
…	Patient no longer willing to participate in domain
…	Clinical team deems that sufficient duration of antibiotic therapy has already been provided

Abbreviations: EOS, early oral switch; IV, intravenous; SAB, *Staphylococcus aureus* bacteremia.

### Interventions

Patients are randomized to EOS (intervention) or continued IV antibiotics (standard of care). The choice of oral antibiotic(s) is at the treating clinician's discretion.

Tables 4 and 5detail a hierarchical list of recommended antibiotics based on the EOS working group's review of the literature. The recommended antibiotics align with antibiotic backbone treatment allocation (eg, oral amoxicillin for those allocated to IV benzylpenicillin). Use of the recommended antibiotic is encouraged but is not mandatory. All participants entering the SNAP trial are randomized at platform entry in a fixed 1:1 ratio to receive EOS or IV treatment. The allocation is revealed once eligibility for EOS domain entry is confirmed. Consent is obtained at platform entry and confirmed at eligibility assessment. Participants enrolled at D7 must receive at least 5 additional days of the allocated treatment strategy (IV antibiotic or EOS). Participants ineligible at D7 are reassessed at D14. Those whose allocation is revealed at D14 must receive a minimum of 12 additional days of the allocated intervention to qualify as having received per-protocol treatment. Those ineligible for EOS at D14 do not participate in the EOS domain.

**Table 4.** Antibiotic Options for EOS in SAB—Dosing, Administration, Pharmacological Properties Principles:
For beta-lactams, maximum doses have been recommended to overcome theoretical issues with drug exposure (bioavailability). Lower doses in specific circumstances have been recommended in the footnotes.Dosing regimens to minimise patient inconvenience have been prioritised, as explained in footnotes.Doses are suggestions only and alternate doses used as standard local practice can be maintained.Contraindications and precautions, including significant drug interactions, and drug toxicities and their required monitoring are not listed and are the responsibility of the prescribing team to review and manage. Some considerations are provided to aid the choice of drug.We have not recommended dose changes for obesity or pregnancy in the setting of EOS (ie, step-down therapy after a period of intravenous therapy/source control/clinical stability). Despite a potential effect of obesity and pregnancy on pharmacokinetics (increased volume of distribution), we do not think this warrants dose adjustment for step-down therapy.With increased creatinine clearance in pregnancy, there is a theoretical concern that the concentration of the antibiotics may not be above the required MIC for a sufficient period of time. However, general practice in obstetric dosing of antibiotics is to dose at the highest end of the dosing range, as is currently planned in the SNAP study.

Drug	Standard Dose	Dose in Renal Impairment^a,b^	Pediatrics	Bio-availability	Fasting	Protein Binding	Pregnancy Category^c^	Half Life	Break Point or ECOFF (June 2023)
Amoxicillin	1 g TDS^d,e^	CrCl 10 to 30 mL/min and CRRT: 1 g 8-hourly. CrCl less than 10 mL/min, HD and PD: 1 g 12-hourly.	25 mg/kg/dose PO (max 2 g) TDS	74%–92%	No	17%–20%	Safe to use all trimesters	1.2–1.5 h	ND
Cefadroxil	1 g BD	CrCl 10–50 mL/min or CRRT: 1 g stat then 500 mg 12-hourly. CrCl less than 10 mL/min: 1 g stat then 500 mg 36-hourly. HD: 1 g stat and 1 g post HD PD: 500 mg 24-hourly.	Not very available for paediatrics 15 mg/kg/dose PO (max 1 g dose) BD	90%	No	20%	Safe to use all trimesters	1.5 h	ND
Cefalexin	1 g TDS^f,g^	CrCl less than 10 mL/min, HD or PD: 1 g 12 hly. CRRT: standard dose.	25 mg/kg/dose PO QDS (max dose 1 g QDS) OR 45 mg/kg/dose PO TDS (max dose 1.5 g TDS) NB TDS dosing is only for children > 12 m of age.	90%	No	10%–19%	Safe to use all trimesters	1 h	ECOFF: 8
Ciprofloxacin PLUS rifampicin (use only in combination)	**Ciprofloxacin:** 750 mg BD	CrCl less than 30 mL/min, HD, PD: 750 mg 24-hourly. CRRT: 250 to 500 mg 12-hourly.	20 mg/kg/dose PO (max 750 mg) BD	70%	No^h^	20%–40%	Avoid in pregnancy	4 h	ECOFF: 1.0
	**Rifampicin:** Weight <60 kg: 600 mg per day; weight >60 kg: 900 mg per day.^i,j^	No change to standard dose.	20 mg/kg/dose PO (max 600 mg) daily	70%–90%	Yes^k^	80%	Reasonable to use in trimester 1 and 2; monitoring required trimester 3 (liver function tests at baseline, Week 1, 2, and 4). May be associated with increased risk of haemorrhagic disorders in newborn.	3 h	BP: 0.06 ECOFF: 0.016
Clindamycin	450 mg TDS^l^	No change to standard dose	10 mg/kg/dose PO (max 450 mg) TDS	55% or 90%	No	94%	Reasonable to use all trimesters	2.4 h	BP: 0.25
Cloxacillin	1 g QDS^m^	No change to standard dose.	50 mg/kg/dose PO (max 500 mg) QDS	32%–50%	No^n^	94%	Reasonable to use all trimesters	0.5 h	0.5
Dicloxacillin	1 g QDS^o,p^	CrCl less than 10 mL/min, HD or PD: 1 g q8h. CRRT: standard dose.	25 mg/kg/dose PO (max 1000 mg) QDS	35%–76%	No^q^	95%	Reasonable to use all trimesters	0.7 h	ND
Doxycycline	100 mg BD	No change to standard dose.	1–2 mg/kg/dose BD PO (max 200 mg per day)	90%	No^r^	93%	Avoid in pregnancy	18 h	ECOFF: 0.5
Flucloxacillin	1 g QDS^s,t^	CrCl less than 10 mL/min, HD or PD: 1 g q8h.CRRT: standard dose.	25 mg/kg/dose (max 1 g) PO QDS	44%–55%	No^u^	95%	Reasonable to use all trimesters	0.75 h	ECOFF: 1
Fusidic acid PLUS rifampicin (use in combination only)	Fusidic acid: 500 mg BD-TDS	No change to standard dose.	**Sodium fusidate (tablets):** 12 mg/kg PO (max 500 mg) TDS. **Fusidic acid (liquid):** 1 m to 18 y: 15 mg/kg PO (max 750 mg) TDS	91%	No	95%–99%	Data scarce in human pregnancy; avoid	8–10 h	BP: 1 ECOFF: 0.5
	**Rifampicin:** Weight <60 kg: 600 mg per day; weight >60 kg: 900 mg per day.^v,w^	No change to standard dose.	20 mg/kg/dose (max 600 mg) daily.	70%–90%	Yes^x^	80%	Reasonable to use in trimesters 1 and 2; monitoring required in trimester 3 (liver function tests at baseline, Week 1, 2 and 4). May be associated with increased risk of hemorrhagic disorders in newborn).	3 h	BP: 0.06 ECOFF: 0.016
Levofloxacin PLUS rifampicin (use in combination only)	**Levofloxacin:** 750 mg daily	CrCl 20–49 mL/min: 750 mg q48h. CrCl < 20 mL/min,HD,PD: 750 mg initial dose; then 500 mg q48h CRRT: 250 mg 24-hourly^y^	10–20 mg/kg/DAY PO (max 500 mg per DAY) in one or 2 divided doses	99%	No	24–38	Avoid in pregnancy	7 h	ECOFF 1
	**Rifampicin:** Weight <60 kg: 600 mg per day; weight >60 kg: 900 mg per day.^z,aa^	No change to standard dose.	20 mg/kg/dose PO (max 600 mg) daily.	70%–90%	Yes^ab^	80%	Safe to use trimester 1 and 2; monitoring required trimester 3 (liver function tests at baseline, Week 1, 2 and 4). May be associated with increased risk of haemorrhagic disorders in newborn).	3 h	BP: 0.06 ECOFF: 0.016
Linezolid	600 mg BD^ac,ad^	CrCl less than 10 mL/min, HD or PD: 600 mg 24-hourly^ae^. CRRT: standard dose.	<12 y: 10 mg/kg/dose PO (max 600 mg) TDS >12 y: 600 mg PO BD	100%	No	30%	No data in human pregnancy; avoid.	5 h	BP: 4 ECOFF: ND
Moxifloxacin PLUS rifampicin (use in combination only)^af^	Moxifloxacin: 400 mg OD	No change to standard dose.	10 mg/kg/dose PO once daily	89%	No	30–50	Avoid in pregnancy	10− 14 h	ECOFF 0.25
	**Rifampicin:** Weight <60 kg: 600 mg per day; weight >60 kg: 900 mg per day.^ag,ah^	No change to standard dose.	20 mg/kg/dose PO (max 600 mg) daily.	70%–90%	Yes^ai^	80%	Safe to use trimester 1 and 2; monitoring required trimester 3 (liver function tests at baseline, Week 1, 2 and 4). May be associated with increased risk of hemorrhagic disorders in newborn).	3 h	BP: 0.06 ECOFF: 0.016
Tedizolid	200 mg OD	No change to standard dose.	Children ≥12 y: 200 mg once daily	91%	No	70%–90%	Little data in pregnancy; should only be used if the benefit justifies the potential risk to the fetus.	12 h	BP: 0.5
Trimethoprim plus sulfamethoxazole (TMP + SMX)	320/1600 mg BD or 160/800 mg TDS	CrCl 26–50 mL/min: normal for 14 d, then 160/800 mg 12-hourly. CrCl 15 to 25 mL/min: normal for 3 d, then 320/1600 mg 24-hourly. For CrCl less than 15 mL/min: avoid use.^aj^	5 mg/kg/dose PO (max 160 mg TMP component) TDS PLUS: folic acid 0.5 mg/kg/dose (max 5 mg) once daily whilst on high dose	70%–90%	No	44/70%	Avoid in first and third trimesters	11 h	BP: 2

Abbreviations: BD, administered twice a day; CrCl, creatinine clearance; CRRT, continuous renal replacement therapy; ECOFF, epidemiological cut-off; EOS, early oral switch; OD, administered once a day; QDS, administered four times a day; SAB, *S. aureus* bacteraemia; TDS, administered three times a day; TGA, Therapeutic Goods Administration, Australia; TMP-SMX, trimethoprim-sulfamethoxazole.

^a^Dose derived from Australian Therapeutic Guidelines: Antibiotic v16, 2019, Sandford Guide and Licensed Product Information from FDA.

^b^HD, hemodialysis, PD, peritoneal dialysis.

^c^Please see Pregnancy appendix for further detail.

^d^Dose derived from POET trial (Partial Oral vs Intravenous Antibiotic Treatment of Endocarditis) [8].

^e^Probenecid (dose: 500 mg if CrCl 60 mL/min or more, 250 mg if CrCl between 60 and 30 mL/min, do not use if CrCL < 30 mL/min) may be co-administered with each dose of beta-lactam to improve drug exposure. Administer with amoxicillin 1 g q6h or 1 g q8h at the discretion of the treating clinician. We recommend giving probenecid with food to prevent nausea.

^f^Clinical efficacy in uncomplicated *S. aureus* bacteremia (SAB) has been demonstrated at a dose of 1 g orally q8h [12].

^g^Probenecid (dose: 500 mg if CrCl 60 mL/min or more, 250 mg if CrCl between 60 to 30 mL/min, do not use if CrCL < 30 mL/min) may be co-administered with each dose of beta-lactam to improve drug exposure. Administer with cefalexin 1 g q6h or 1 g q8h at the discretion of the treating clinician. We recommend giving probenecid with food to prevent nausea.

^h^Ledergerber et al, Effect of standard breakfast on drug absorption and multiple-dose pharmacokinetics of ciprofloxacin [24].

^i^Doses above 600 mg per day should be divided into two doses.

^j^Use with caution in liver disease—can cause hepatotoxicity.

^k^Ideally, administer 30 minutes before or two hours after a meal.

^l^For oral administration 450 mg is the maximum dose licensed by the TGA. Clindamycin dosed 8-hourly showed significantly longer bactericidal activity against *S. aureus* when compared to 12-hourly regimens, (87.5% to 100% vs 49.6% to 77.1%, *P* < .001) [25].

^m^Probenecid (dose: 500 mg if CrCl 60 mL/min or more, 250 mg if CrCl between 60 to 30 mL/min, do not use if CrCL < 30 mL/min) may be co-administered with each dose of beta-lactam to improve drug exposure. Administer with cloxacillin 1 g q6h at the discretion of the treating clinician. We recommend giving probenecid with food to prevent nausea.

^n^Product information advises administration in the fasting state to maximise bioavailability, but this may make adherence difficult. Data that show decreased clinical efficacy when administered with food are lacking. We have recommended a high dose to optimize drug concentrations if administration in the fasted state is not possible.

^o^Dose derived from POET trial [8].

^p^Probenecid (dose: 500 mg if CrCl 60 mL/min or more, 250 mg if CrCl between 60 to 30 mL/min, do not use if CrCL < 30 mL/min) may be co-administered with each dose of beta-lactam to improve drug exposure. Administer with dicloxacillin 1 g q6h or 1 g q8h at the discretion of the treating clinician. We recommend giving probenecid with food to prevent nausea.

^q^Product information advises administration in the fasting state to maximise bioavailability, but this may make adherence difficult. Data that show decreased clinical efficacy when administered with food are lacking. We have recommended a high dose to optimise drug concentrations if administration in the fasted state is not possible.

^r^Taking doxycycline on an empty stomach can cause nausea.

^s^Clinical efficacy in uncomplicated SAB has been demonstrated at a dose of 1 g orally q8h [12].

^t^Probenecid (dose: 500 mg if CrCl 60 mL/min or more, 250 mg if CrCl between 60 to 30 mL/min, do not use if CrCL < 30 mL/min) may be co-administered with each dose of beta-lactam to improve drug exposure. Administer with flucloxacillin 1 g q6h or 1 g q8h at the discretion of the treating clinician [26].

^u^Although the product information recommends administration in the fasting state to maximise bioavailability, administration with food is unlikely to reduce efficacy in most situations [27]. We have recommended a high dose of flucloxacillin to optimise drug concentrations.

^v^Doses above 600 mg per day should be divided into two doses.

^w^Use with caution in liver disease—can cause hepatotoxicity.

^x^Ideally, administer 30 minutes before or two hours after a meal.

^y^Malone RS et al, Pharmacokinetics of levofloxacin and ciprofloxacin during continuous renal replacement therapy in critically ill patients [28].

^z^Doses above 600 mg per day should be divided into 2 doses.

^aa^Use with caution in liver disease—can cause hepatotoxicity.

^ab^Ideally, administer 30 minutes before or two hours after a meal.

^ac^Risk of haematological toxicity increases with use beyond 14 d [29].

^ad^Pyridoxine 50mg-100 mg/day to prevent or delay anaemia can be considered if using linezolid for > 7 d; evidence for benefit conflicting [30].

^ae^The optimal dose of linezolid in renal impairment is unknown, alternative doses include 300 mg 12-hourly or 600 mg 12-hourly. Patients are at an increased risk of thrombocytopenia if continued on 600 mg 12-hourly in the setting of renal impairment. Therapeutic drug monitoring aiming for a trough concentration between 2 and 7 mg/L is recommended for patients on linezolid with renal impairment [31].

^af^Rifampicin may reduce serum concentrations of moxifloxacin, though the clinical significance of this interaction remains uncertain. Consider using another quinolone in combination with rifampicin.

^ag^Doses above 600 mg per day should be divided into 2 doses.

^ah^Use with caution in liver disease—can cause hepatotoxicity.

^ai^Ideally, administer 30 minutes before or 2 hours after a meal.

^aj^Sulfamethoxazole can cause pancreatic insulin release, resulting in clinically significant hypoglycaemia, particularly in patients with renal impairment, receiving high doses, or concomitantly taking a sulfonylurea [32].

**Table 5.** Hierarchy of Recommended Oral Antibiotics for EOS by Silo (ie Susceptibility of *S. aureus*)

Silo	Recommended Oral Antibiotic According to Allocated Backbone Domain					
	Adult		Pregnancy		Pediatric	
PSSA	Benzylpenicillin	(Flu)cloxacillin	Benzylpenicillin	(Flu)cloxacillin	Benzylpenicillin	(Flu)cloxacillin
	Amoxicillin (Flu/di)cloxacillin Cefalexin/cefadroxil Linezolid	(Flu/di)cloxacillin Amoxicillin Cefalexin/cefadroxil Linezolid	Amoxicillin (Flu/di)cloxacillin Cefalexin/cefadroxil	(Flu/di)cloxacillin Amoxicillin Cefalexin/cefadroxil	Amoxicillin Cefalexin/cefadroxil (Flu/di)cloxacillin Linezolid	Cefalexin/cefadroxil (Flu/di)cloxacillin Amoxicillin Linezolid
MSSA	(Flu)cloxacillin	Cefazolin	(Flu)cloxacillin	Cefazolin	(Flu)cloxacillin	Cefazolin
	(Flu/di)cloxacillin Cefalexin/cefadroxil Linezolid	Cefalexin/cefadroxil (Flu/di)cloxacillin Linezolid	(Flu/di)cloxacillin Cefalexin/cefadroxil	Cefalexin/cefadroxil (Flu/di)cloxacillin	Cefalexin/cefadroxil (Flu/di)cloxacillin Linezolid	Cefalexin/cefadroxil (Flu/di)cloxacillin Linezolid
MRSA	Vancomycin/daptomycin	Vancomycin/daptomycin + cefazolin	Vancomycin/daptomycin	Vancomycin/daptomycin + cefazolin	Vancomycin/daptomycin	Vancomycin/daptomycin + cefazolin
	Linezolid Fluoroquinolone + rifampicin TMP-SMX Fusidic acid + rifampicin	Linezolid Fluoroquinolone + rifampicin TMP-SMX Fusidic acid + rifampicin	Clindamycin TMP-SMX^a^	Clindamycin TMP-SMX^a^	TMP-SMX Linezolid Fluoroquinolone + rifampicin Fusidic acid + rifampicin	TMP-SMX Linezolid Fluoroquinolone + rifampicin Fusidic acid + rifampicin

Site PIs and treating clinicians are encouraged, but not mandated, to select the highest antibiotic on this list which is appropriate for a given patient.

Abbreviations: EOS, early oral switch; MRSA, methicillin-resistant *Staphylococcus aureus*; MSSA, methicillin- susceptible *Staphylococcus aureus*; PSSA, penicillin-susceptible *Staphylococcus aureus*; TMP-SMX, trimethoprim-sulfamethoxazole.

^a^TMP-SMX only suitable during the second trimester. Avoid in first and third trimester.

After study-mandated minimum treatment durations, the antibiotic choice, route, and total duration of treatment are at clinician's discretion. The assigned treatment strategy is continued unless a participant dies or withdraws from the domain or the treating clinician deems continued participation no longer in their best interests.

### Participant Timeline

As indicated in Table 6, minimal assessments or investigations are required on top of routine care, apart from the eligibility assessments, and data collection.

**Table 6.** Domain-specific Schedule of Visits and Follow-up

Platform Day	Day 1	Day 7 (±2)	Day 14 (±2)	Acute D/C	Total D/C^a^	Day 21 (±3)	Day 28 (±3)	Day 42 (±3)
Consent	X	…	…	…	…	…	…	…
Eligibility assessment for EOS	…	X	X	…	…	…	…	…
Allocation reveal if eligible	…	X	X	…	…	…	…	…
Data on antibiotics ± adherence	…	…	X^b^	X	X	…	X^b^	X^b^
Check clinical progress^c^	…	X	X	…	…	X	X	X

Abbreviations: EOS, early oral switch; OPAT, outpatient antibiotic treatmen; HITH, Hospital in the home.

^a^Total discharge means the end of the total index hospital admission, which includes both inpatient and OPAT/HITH/rehab stay.

^b^If participant is discharged at this timepoint, collect data from medical records where possible, or phone call to the participant.

^c^For those whose antibiotic treatment is ongoing, check that treating team have spoken to or seen the patient, to assess symptoms of infection, oral antibiotic adherence and adverse effects.

### Endpoints

The primary endpoint for the EOS domain is the SNAP core primary endpoint: 90-day all-cause mortality. This will be captured via case report forms, reviewing hospital records, contact with family or primary care provider, and, where available, linked to nationally available data such as death registries. Death certificates will not be reviewed. Secondary endpoints specific to IV antibiotics and EOS are assessed (Table 7). In addition, SNAP will report 2 different “desirability of outcome ranking” (DOOR) analyses as part of the Core Protocol. Although not specific to the EOS domain, patient centered outcomes such as treatment failure, adverse events, functional capacity, and length of stay will therefore be measured.

**Table 7.** Primary and Secondary Endpoints for the EOS Domain

Endpoint	Endpoint Measure
Primary	All-cause mortality 90 d after platform entry.
Secondary	1. Number of days of IV antibiotic therapy in the total index hospitalization (which includes OPAT), starting from platform entry, for those surviving until hospital discharge
	2. Number of days alive and free of antibiotics by Day 42 from platform entry (a) For all antibiotics (b) For IV antibiotics
	3. Peripherally inserted central catheter (PICC)/other central venous catheter complications requiring line removal, during the total index hospitalisation (which includes OPAT), starting from platform entry (a) This outcome will be collected at total index hospital/OPAT discharge as a Y/N question. It will include any of the following: catheter-related blood stream infection; exit site infection; catheter-related superficial or deep venous thrombosis/thrombophlebitis, catheter blockage. It will NOT include PICC line rupture, leakage, displacement, or splitting unless it results in or occurs in addition to one of the above events.
	4. Clinician-initiated change in treatment strategy from allocated EOS domain intervention (eg, changed to IV antibiotics when allocated to oral antibiotics or vice versa) from reveal of EOS allocation until platform day 28 due to an adverse event deemed by the treating doctor/team to be of sufficient severity to change strategy.
	5. Clinician-initiated change in treatment strategy from allocated EOS domain intervention (eg, changed to IV antibiotics when allocated to oral antibiotics or vice versa) from reveal of EOS allocation until platform day 28 due to presumed lack of efficacy of strategy according to the treating doctor/team.

Abbreviations: EOS, early oral switch; IV, intravenous; OPAT, outpatient antibiotic treatment; N, no; Y, yes.

### Analysis

The primary objective for this domain is to assess the non-inferiority of EOS compared with IV treatment. The primary analysis population is the intention to treat (ITT) population. We will also report results in the per protocol population (PPP). The PPP for treatment in the EOS domain is participants enrolled at D7 who receive ≥ 5 days of the randomized antibiotic route during platform days 8–14, or if enrolled at D14, receive ≥ 12 days of the randomized antibiotic route during platform days 15–28 inclusive. Participants who deviate from the allocated treatment strategy and duration (eg, a participant allocated to oral antibiotics at D14 who receives a period of IV treatment for intercurrent infection in the following 12 days) are not considered in the PPP. Similarly, participants who are allocated to IV administration, yet request discharge from hospital on oral antibiotics resulting in a cessation of IV antibiotics prior to the PPP mandated durations, will not be considered in the PPP. Participants who die within these timepoints are also considered to be in the per protocol population.

The primary estimate is the effect of the intervention (EOS) compared to the domain control (IV antibiotics) on the probability of the primary endpoint in platform-eligible participants in the adult population. Non-inferiority has been defined as an odds ratio (OR) of <1.2 for the primary endpoint, where an OR > 1 represents an increase in mortality compared to the domain control. This boundary was chosen because the trial committees felt that a clinically minimal important difference should numerically correspond to a 3% absolute difference if the expected mortality rate in the control group was 15%. If the control mortality rate is lower than 15%, the use of a ratio scale ensures that the corresponding acceptable absolute difference will equally become proportionately smaller. This margin is considerably less than most studies, where the absolute difference is often in the order of 10% [26]. The non-inferiority OR for an absolute difference of 10% (15% baseline, 25% intervention) is 1.67. Statistical triggers for concluding non-inferiority are if the posterior probability that OR < 1.2 is >.99 and conversely for futility of the non-inferiority objective if the probability that OR < 1.2 is < .01.

### Data Collection and Management

The EOS domain requires specific additional data collection, particularly total antibiotic use from index hospitalisation to platform day 42, including route of administration and related complications. Reasons for changes to the allocated treatment strategy are included as secondary endpoints: clinical failure attributed to the allocated strategy, or adverse reactions severe enough to warrant a change in strategy despite study allocation.

Assessment of treatment adherence is collected on platform days 14 and 28. Adherence is assessed via medication administration records and/or participant questionnaire by identifying the number of days in the past week that the participant missed at least 1 dose of antibiotics (0–1, 2–3, or >3).

Venous catheter-related complications are captured until total index hospital discharge (including time spent under outpatient parenteral antibiotic therapy (OPAT) supervision). This includes catheter-related bloodstream infection, exit site infection, blockage, and superficial or deep venous thrombosis/thrombophlebitis.

Follow-up will be performed via review of medical notes and medication administration records, or by participant telephone interview depending on whether the participant is still an inpatient at the data collection points (Table 6).

## DISCUSSION

SNAP is now the largest *S. aureus* bacteremia treatment trial ever undertaken [23]. The EOS domain will substantially add to evidence regarding the safety and efficacy of partial oral antibiotic therapy for the treatment of both complicated and uncomplicated SAB. Although some clinicians may already be comfortable with EOS in some SAB scenarios, we aim to demonstrate effectiveness of this strategy across the spectrum of SAB disease. The EOS strategy has the potential to benefit both patients—in terms of convenience, acceptability, and risk reduction—and healthcare systems, due to reduction in the costs, resource utilisation, and length of stay related to prolonged IV therapy.

Mortality is a clear binary endpoint and can be compared and used across trials. Prior engagement with patients and representatives highlighted mortality as the patient-preferred endpoint. Composite outcomes like DOOR provide an alternative endpoint measure for SAB trials. We have included 2 versions of a DOOR as core secondary endpoints for the Core Protocol; however, for the EOS domain other key secondary endpoints include complications of IV therapy and a clinical decision to change the treatment pathway.

Data collection will be healthcare-focused and pragmatic. We aim to explore patient-reported health-related quality of life through a health economic analysis. For some regions, EQ-5D-5L will be collected as well as the functional bloodstream score, although this will not specifically address the patient's perception of benefits or drawbacks of the route of administration. Another limitation is that we will not be capturing side effects related to oral therapy (such as nausea). This was not included in order to simplify the patient activities needed to participate in the trial. Clinical experience suggests that patients prefer oral over prolonged IV treatment, and there is scope for future inclusion of a qualitative measure, or further qualitative studies in this area.

### Trial Status

**Figure 1.**
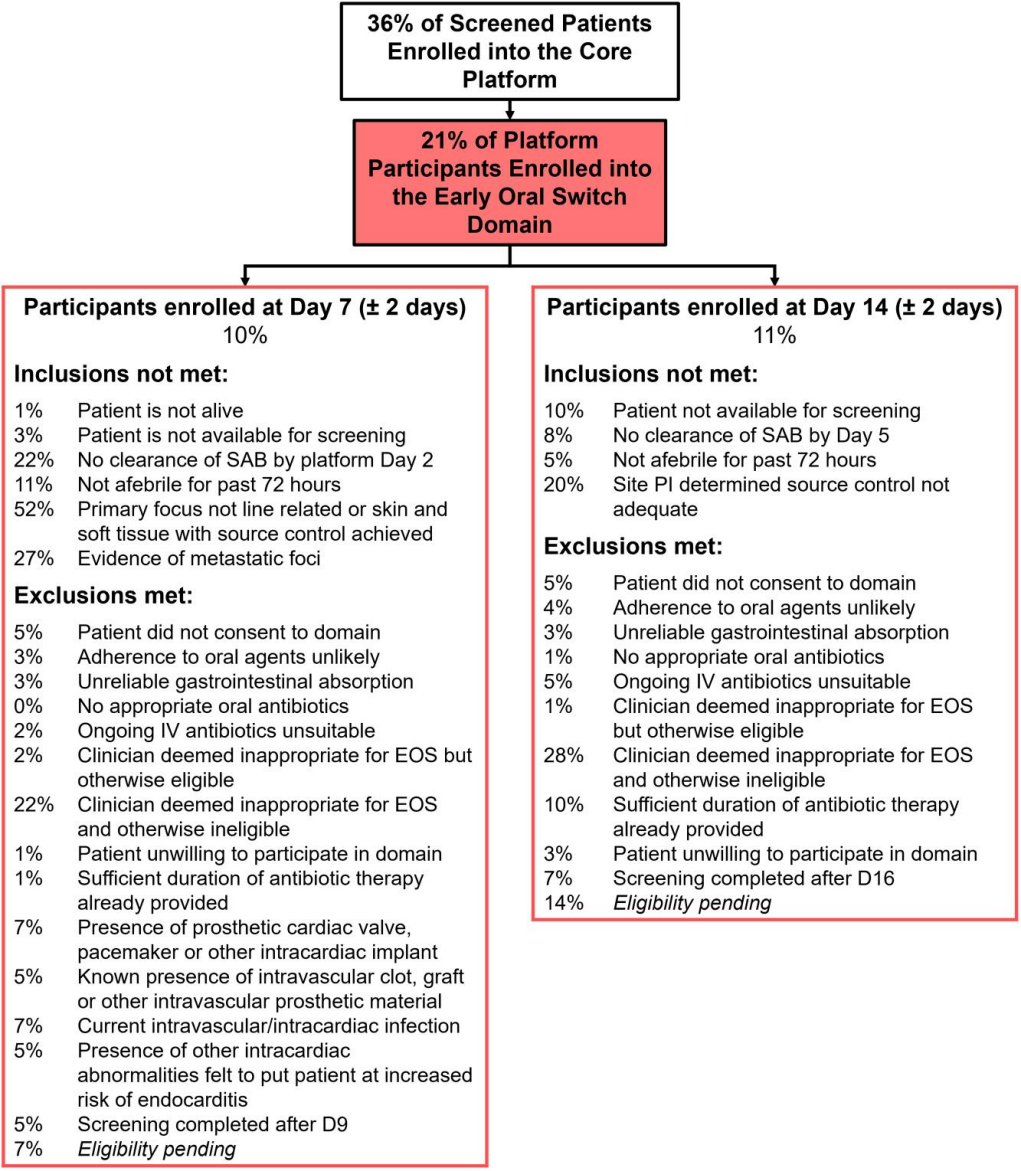
SNAP CONSORT as of 21 August 2023. Abbreviations: CONSORT, Consolidated Standards of Reporting Trials; EOS, early oral switch; IV, intravenous; PI, principal investigator; SAB, *S. aureus* bacteraemia; SNAP, *S. aureus* Network Adaptive Platform.

SNAP opened in February 2022 and is recruiting over 100 participants per month at the time of writing. As of August 2023, 21% of platform participants have entered the EOS domain (10% at D7% and 11% at D14), This is lower than our initial estimate of 45% and recruitment has been slower than expected but is increasing overall as more sites join the trial. The proportion of uncomplicated SAB patients entering the EOS domain (125 of total 3384 SAB cases screened, 4%) is similar to that achieved by the recent SABATO trial (4%) [9], although the overall proportion of screened patients entering the EOS domain (250/3213, 8%) is higher, due to the inclusion of patients with complicated SAB being enrolled at the later time point. Figure 1 outlines reasons for non-inclusion or exclusion from the EOS domain. The inclusion/exclusion criteria for the EOS domain are deliberately conservative. The authors are aware that opinions vary regarding partial oral therapy for SAB, with some clinicians embracing the EOS ethos and others being strong proponents of traditional IV standard of care. The protocol attempts to encompass both clinical viewpoints. The most common reasons for *non-inclusion* of participants at D7 were inadequate source control, or metastatic infection; at D14 inadequate source control was most commonly cited. The most common reason for *exclusion* was the criterion, “clinician deems not appropriate for EOS’. However, if we discount those participants who were also ineligible for other reasons, only 2% of patients at D7% and 1% at D14 were excluded purely because of clinician hesitancy. Investigators are asked to provide a reason for choosing this exclusion criterion in order to investigate the causes of clinician hesitancy. Although the authors and trial steering committee have equipoise for the two interventions, this is not universal among clinicians. Mortality associated with SAB in adults is high, and this knowledge may lead a conservative approach with respect to antibiotic route and duration. Evidence exists to reassure clinicians that EOS is safe and effective (Tables 1 and 2). In direct contrast, paediatricians are hesitant to risk potentially randomising children to longer IV therapy than clinically necessary; placement of a PICC and attendant prolonged hospital stay is not seen as desirable in children. Consequently there has been lower recruitment to the paediatric arm of the EOS domain.

At D7, 52% of participants were recorded as having inadequate source control; this remained a significant cause of non-inclusion at D14 (20%). One of the additional barriers to enrolment at D14 has been the failure to achieve sterile blood cultures by D5, occurring in 8% of those assessed. Prolonged bacteraemia usually results from inadequate source control, and there is evidence that this is associated with worse outcomes [33, 34]. Planned analysis from SNAP will provide insight into this group.

Other barriers to recruitment of adults to date have included concerns about poor adherence to oral antibiotics. Resource constraints make directly observed therapy unrealistic in most settings. Re-purposing existing OPAT services to support adherence to oral antibiotics (complex outpatient antibiotic treatment—“COPAT”) and to facilitate EOS domain enrolment (eg, phone calls to participants after hospital discharge) has been successful at some participating sites.

Early engagement with treating teams, eg orthopaedic surgeons, is an important strategy for recruitment. This allows objections and logistics to be addressed prior to eligibility assessment. Group discussions and consensus (eg departmental meetings) can further improve willingness to enroll. Ongoing clinician education regarding SNAP, its protocols, the literature surrounding EOS, and pharmacokinetic/dynamic aspects of antibiotic use can also contribute to improving clinician engagement and enrolment.

Equally important is engagement and regular communication with the participant. It can be up to 16 days between a participant consenting to the platform, and being assessed for eligibility for the EOS domain. Conveying the idea of clinical equipoise in layperson terms may provide reassurance and improve the participant's willingness to participate in the domain. Consent for the EOS domain is reconfirmed before assessing eligibility; around 10% of platform patients do not give consent for this domain.

A limitation for recruitment to the EOS domain is a whole-platform exclusion for patients identified or approached more than 72 hours after the index blood culture collection. This 72-hour cut-off is necessary to ensure the validity of data in other domains; however, many patients who may otherwise be eligible for the EOS domain are excluded as a result. The data pertaining to numbers excluded are not shown in Figure 1, which solely explores those already in the platform. As of August 2023, 619 patients were excluded from the platform solely because they did not meet the 72-hour cut-off; this represents 19% of those screened for the trial who may have contributed to the EOS domain. These patients were instead recruited to an observational registry.

## CONCLUSIONS

The SNAP trial is an innovative approach to studying the management of SAB, allowing multiple treatments to be studied in parallel. The EOS domain aims to determine whether EOS is non-inferior to traditional prolonged IV treatment. Conventional teaching means using oral antibiotics to complete a SAB treatment course remains an unfamiliar and intimidating idea for many clinicians who treat adults. Barriers to enrollment in this domain include inadequate source control and persistent bacteremia, clinician hesitancy, and concerns about adherence. We hope to contribute high-quality evidence to the debate on the safety and efficacy of oral antibiotics in the treatment of SAB and eventually to be part of a paradigm shift in the management of this serious and common condition.

## Supplementary Data

Supplementary materials are available at *Clinical Infectious Diseases* online. Consisting of data provided by the authors to benefit the reader, the posted materials are not copyedited and are the sole responsibility of the authors, so questions or comments should be addressed to the corresponding author.

## Supplementary Material

ciad666_Supplementary_Data

## Notes

***Acknowledgments.*** The SNAP trial is an international platform with a large number of contributors. A. L. Goodman and G. Walls co-chair the domain-specific working group who conceived of and wrote the protocol for this part of the SNAP trial and are thus joint lead senior authors for this article. D. de Kretser was supervised by G. Walls and subsequently A. L. Goodman and worked across centres to develop the protocol. D. de Kretser, A. L. Goodman, and G. Walls wrote the first draft of the article and collated all further input and responses. A. L. Goodman is the corresponding author. All SNAP EOS Domain-Specific Working Group authors conceived of the idea and contributed to the design and final article preparation. S. Y. C. Tong and J. S. Davis were instrumental in developing the original concept of the SNAP platform design and are senior SNAP platform leads and should be considered additional senior authors. Concepts, protocol design, and writing were improved, edited, and reviewed through the SNAP Trial Global Trial Steering Committee (as composed at the time of drafting and writing) and authors are listed, although not repeated. J. Marsh, S. Y. C. Tong, J. S. Davis, A. L. Goodman, G. Walls, S. C. Morpeth, M. Hensgens, J. Mora, D. Yahav, A. McGlothlin, M. P. Cheng contribute to both the Global Trial Steering Committee and the EOS Domain-Specific Working Group, so their names are not repeated. Staphylococcus aureus Network Adaptive Platform (SNAP) Study Group members listed may differ from the group listed on other manuscripts and are specific to this article. These are collaborating authors who contributed to the design of the protocol and funding applications in their regions though not directly to the writing of this article. The authors would like to thank the DSMC and the consumer reference groups and public patient involvement (PPI) groups in all regions for their input into protocol design. L. Whiteway and J. Bostock are consumer/PPI representatives.

***Financial support*.** The SNAP trial, including the EOS domain, has funding from several national health research funding bodies: the Australian National Health and Medical Research Council (Australia) (NHMRC grant number 2014900), the Canadian Institutes of Health Research (grant number 451092), the New Zealand Health Research Council of New Zealand (grant number 20/344), the National Institute for Health and Care Research (United Kingdom) (NIHR [133719], the views expressed are those of the author(s) and not necessarily those of the UK NIHR or the Department of Health and Social Care), the Medical Research Council (MC_UU_00004/05) and the Singapore National Medical Research Council (Singapore) (CTGIIT21nov-0002). The pediatric EOS domain has additional funding from the Starship Foundation (New Zealand) (ASF2144_WEBB) and the NHMRC (grant number 1184238). J. A. R. receives funding from the Australian National Health and Medical Research Council (Australia) for a Center of Research Excellence (grant number APP2007007) and an Investigator Grant (grant number APP2009736), as well as an Advancing Queensland (Australia) Clinical Fellowship, A. C. B. also reports National Health and Medical Research Council funding 1175509 (fellowship for my salary). G. W. also reports support for this work from Counties Manukau Tupu Health Fund (SNAPPY) and Te Niwha Infectious Diseases Research Fund (PR-O-SNAP).

***The SNAP Trial Group.*** Nick Anagnostou (Flinders Medical Centre, Adelaide, South Australia, Australia); Sophia Acrhuleta (Division of Infectious Diseases, National University Hospital, Singapore); Eugene Athan (School of Medicine, Deakin University, Victoria, Australia); Lauren Barina (Department of Infectious Diseases University of Melbourne, Peter Doherty Institute for Infection and Immunity, Melbourne, Australia); Emma Best (Department of Paediatrics, University of Auckland, New Zealand; Department of Infectious Disease, Starship Children’s Hospital, Auckland, New Zealand); Katie Brett (School of Medicine and Public Health and Hunter Medical Research Institute, University of Newcastle, Newcastle, Australia); Hannah Burden (Aotearoa Clinical Trials, Auckland, New Zealand); Peter Daley (Eastern Health Region, St. John's, NL, Canada); Jane Davies (Menzies School of Health Research, Charles Darwin University, Darwin, Northern Territory, Australia); Partha P De (National Centre for Infectious Diseases, Singapore, Department of Infectious Diseases, Tan Tock Seng Hospital, Singapore); Yael Dishon-Benattar (Faculty of Social Welfare and Health Sciences, University of Haifa, Israel); Katie Flanagan (Clinical Infectious Disease Service, Launceston General Hospital, Tasmania, Australia); Jennifer Grant (Faculty of Medicine, University of British Columbia, Vancouver, BC, Canada); Dan Gregson (Departments of Pathology and Laboratory Medicine, University of Calgary, Canada); Kate Grimwade (Toi Te Ora Public Health and Bay of Plenty District Health Board, Tauranga, New Zealand); James Hatcher (Department of Microbiology, Virology and Infection Control, Great Ormond Street Hospital, London WC1N 3JH, UK); Andrew Henderson (Department of Infectious Diseases, Princess Alexandra Hospital, Brisbane, Australia); Dina Jankovic (Centre for Health Economics, University of York, York, UK); Jennie Johnstone (Department of Laboratory Medicine and Pathobiology, University of Toronto, Toronto, Ontario, Canada; Public Health Ontario, Toronto, Ontario, Canada.); I Russel Lee (Yong Loo Lin School of Medicine, Singapore; Lee Kong Chian School of Medicine, Singapore); Ka Lip Chew (Yong Loo Lin School of Medicine, Singapore; Lee Kong Chian School of Medicine, Singapore); Martin Llewelyn (Department of Global Health and Infectious Diseases, Brighton and Sussex Medical School, Brighton, United Kingdom); Anne-Grete Martson (Institute of Systems, Molecular and Integrative Biology, University of Liverpool); Colin McArthur (Department of Critical Care Medicine, Auckland City Hospital, New Zealand); Diana McNeil (Department of Infectious Diseases, Middlemore Hospital, Auckland, New Zealand); Sarah Metcalf (Department of Infectious Diseases, Canterbury District Health Board, Christchurch, New Zealand); Clare Nourse (School of Medicine, University of Queensland, Brisbane, Australia, Infection Management and Prevention Service, Queensland Children’s Hospital, Children’s Health); Matthew O’Sullivan (Department of Internal Medicine and Radboud Centre for Infectious Diseases, Radboud University Medical Center, 6525 GA Nijmegen, The Netherlands); Lina Petrella (Divisions of Infectious Diseases and Medical Microbiology, McGill University Health Centre, Montreal, Canada); Sarah Pett (Medical Research Council Clinical Trials Unit, University College London, London, United Kingdom); Benjamin A Rogers (Monash University (including Australian and New Zealand Intensive Care Research Centre); Clayton, 3800, Australia, Australia); James Sim (Victorian Infectious Diseases Service, The Royal Melbourne Hospital, at the Peter Doherty Institute for Infection and Immunity, Melbourne, Australia; School of Medicine and Public Health and Hunter Medical Research Institute, University of Newcastle, Newcastle, Australia); Marta O Soares (Centre for Health Economics, University of York, York, UK); Neil Stone (University College London Hospitals NHS Foundation Trust, London, UK); Robert Tilley (Department of Microbiology, University Hospitals Plymouth NHS Trust, Plymouth, UK); Rebecca Turner (Medical Research Council Clinical Trials Unit, University College London, London, United Kingdom); Viliame Tutone (Department of Clinical Research, London School of Hygiene & Tropical Medicine, London, United Kingdom); Jonathan Underwood (School of Medicine, Cardiff University, University Hospital of Wales, Heath Park, Cardiff CF14 4XN, United Kingdom); Lesley Voss (Department of Infectious Disease, Starship Children’s Hospital, Auckland, New Zealand); Rachel H Webb (Department of Infectious Disease, Starship Children’s Hospital, Auckland, New Zealand); Heather Wilson (Canberra Hospital); Terence Wuerz (University of Manitoba, Winnipeg, Manitoba, Canada).
