# Supplementary material for: Early Oral Antibiotic Switch in Staphylococcus aureus Bacteraemia: The Staphylococcus aureus Network Adaptive Platform (SNAP) Trial Early Oral Switch Protocol
Source: Clin Infect Dis. 2023 Oct 31;79(4):871–87. doi: 10.1093/cid/ciad666 (PMC11478773; doi:10.1093/cid/ciad666)
Supplement: ciad666_Supplementary_Data [file ciad666_supplementary_data.docx]

# Title: Early oral antibiotic switch in *Staphylococcus aureus* bacteraemia: The *Staphylococcus aureus* Network Adaptive Platform (SNAP) Trial Early Oral Switch Protocol

# Supplementary Appendix

**Staphylococcus aureus Network Adaptive Platform (SNAP) Study Group members**

**Collaborating authors who contributed to the design and funding applications in their regions**

Nick Anagnostou^45^, Sophia Acrhuleta^46^, Eugene Athan^47^, Lauren Barina^2^, Emma Best^48,49^, Katie Brett^27^, Hannah Burden^50^, Peter Daley^51^, Jane Davies^13^, Partha P De^32^, Yael Dishon-Benattar^52^, Katie Flanagan^53^, Jennifer Grant^54^, Dan Gregson^55^, Kate Grimwade^56^, James Hatcher^57^, Andrew Henderson^58^, Dina Jankovic^59^, Jennie Johnstone^60^, I Russel Lee^33,34^, Ka Lip Chew^33,34^, Martin Llewelyn^61^, Anne-Grete Martson^62^, Colin McArthur^63^, Diana McNeil^22^, Sarah Metcalf^64^, Clare Nourse^65,66^, Matthew O’Sullivan^30^, Lina Petrella^5^, Sarah Pett^1^, Benjamin A Rogers^7^, James Sim^33,34^, Marta O Soares^59^, Neil Stone^18^, Robert Tilley^67^, Rebecca Turner^1^, Viliame Tutone^22^, Jonathan Underwood^68^, Lesley Voss^49^, Rachel H Webb^49^, Heather Wilson^69^, Terence Wuerz^70^

45. Flinders Medical Centre, Adelaide, South Australia, Australia

46. Division of Infectious Diseases, National University Hospital, Singapore

47. School of Medicine, Deakin University, Victoria, Australia

48. Department of Paediatrics, University of Auckland, New Zealand

49. Department of Infectious Disease, Starship Children’s Hospital, Auckland, New Zealand

50. Aotearoa Clinical Trials, Auckland, New Zealand

51. Eastern Health Region, St. John's, NL, Canada

52. Faculty of Social Welfare and Health Sciences, University of Haifa, Israel

53. Clinical Infectious Disease Service, Launceston General Hospital, Tasmania, Australia

54. Faculty of Medicine, University of British Columbia, Vancouver, BC, Canada

55. Departments of Pathology and Laboratory Medicine, University of Calgary, Canada

56. Toi Te Ora Public Health and Bay of Plenty District Health Board, Tauranga, New Zealand

57. Department of Microbiology, Virology and Infection Control, Great Ormond Street Hospital, London WC1N 3JH, UK

58. Department of Infectious Diseases, Princess Alexandra Hospital, Brisbane, Australia

59. Centre for Health Economics, University of York, York, UK

60. Department of Laboratory Medicine and Pathobiology, University of Toronto, Toronto, Ontario, Canada; Public Health Ontario, Toronto, Ontario, Canada.

61. Department of Global Health and Infectious Diseases, Brighton and Sussex Medical School, Brighton, United Kingdom

62. Institute of Systems, Molecular and Integrative Biology, University of Liverpool

63. Department of Critical Care Medicine, Auckland City Hospital, New Zealand

64. Department of Infectious Diseases, Canterbury District Health Board, Christchurch, New Zealand

65. School of Medicine, University of Queensland, Brisbane, Australia

66. Infection Management and Prevention Service, Queensland Children’s Hospital, Children’s Health Queensland Hospital and Health Service, Brisbane, Australia

67. Department of Microbiology, University Hospitals Plymouth NHS Trust, Plymouth, UK

68. School of Medicine, Cardiff University, University Hospital of Wales, Heath Park, Cardiff CF14 4XN, United Kingdom

69. Canberra Hospital

70. University of Manitoba, Winnipeg, Manitoba, Canada

## Methods

In the overall SNAP platform, participants are currently recruited to one, two or all of three current domains: the antibiotic backbone domain, the adjunctive treatment domain, and the EOS domain. Participants are enrolled into the antibiotic backbone domain within 72 hours of index blood culture collection and are randomised to initial IV therapy according to the antibiotic susceptibility of their *S. aureus* isolate. For penicillin-susceptible isolates (PSSA), participants are randomised to IV benzylpenicillin or (flu)cloxacillin. For methicillin-susceptible but penicillin-resistant (MSSA), participants are randomised to IV (flu)cloxacillin or IV cefazolin, and for methicillin-resistant (MRSA), participants who are already receiving vancomycin or daptomycin are randomised to additionally receive or not receive 7 days of adjunctive IV cefazolin. All these IV treatments would typically be for at least 14 days unless the participant is enrolled to the EOS domain.

In the SNAP trial ‘complicated’ SAB is defined as participants with one or more of the following:

i. An implanted intravascular prosthesis or endovascular device

ii. Day 2 (from platform entry) blood cultures positive

iii. Fever (any temperature 37.8 C or above on platform day 2)

iv. Evidence of deep seated (i.e., not just line related or skin and soft tissue related) or metastatic infection. This includes evidence of endocarditis.

Although the final categorisation of complicated and uncomplicated may relate to a final outcome such as attributable mortality or recurrent infection within 12 weeks, it can be related to the presence of a site of infection remote from the primary focus, such as endocarditis, osteomyelitis (haematogenous seeding), thrombophlebitis or abscess (extension of the infection) (35). These assessments influence the overall management plan in SAB (2).

The key challenges in accurately comparing these two interventions are ensuring that both adherence and default duration of treatment are consistent between the two arms. For example, monitoring treatment adherence is often more straightforward in patients receiving prolonged IV therapy. These patients are usually closely followed by an OPAT team and often have a healthcare provider administering the antibiotics. Adherence is therefore closely monitored and non-adherence quickly identified. Fortunately, many sites now also use their OPAT services (sometimes called ‘COPAT’ or Complex OutPatient Antibiotic Therapy) to monitor patients on long-term oral antibiotics.

Assessment of adherence in those discharged on oral antibiotics is potentially lower due to reliance on self-administration and participant reports. Adherence to oral antibiotics is affected by the frequency of administration, especially when combined with restrictions around food, and the duration of treatment. The clinician may be naturally inclined to a shorter total IV treatment course, as they will be aware of the inconvenience and practical implications of such treatment, whereas a clinician may extend a well-tolerated oral treatment course in an effort to minimise any risk of a disease recurrence or poor outcome.

## Trial Statistics

A statistical simulation study was performed for the SNAP platform assuming a maximum sample size of 7000 participants (6000 adults and 1000 children). The simulation study demonstrated that SNAP has adequate power to assess these outcomes and detect non-inferiority based on a moderate effect size. This assumes that 10% of all participants will be eligible and enrolled into the EOS domain at the Day 7 timepoint, and an additional 45% will be eligible and randomised by Day 14. The mortality rates assumed for the simulation study for adults and children in each silo were PSSA (adults 15%, children 2.2%), MSSA (15%, 2.2%), and MRSA (20%, 3.5%). We accounted for baseline mortality rates being different for those eligible at day 7 (7% for PSSA and MSSA, 9.7% for MRSA), those eligible at day 14 (15% for PSSA and MSSA, 20% for MRSA), and those never reaching eligibility (e.g., died prior) or not eligible by day 14 (16.8% for PSSA and MSSA, 22.3% for MRSA). For the ‘null’ scenario, where the odds ratio was set to 1.2 for the non-inferiority hypotheses in the backbone antibiotic and EOS domains (and 1.0 for the superiority hypotheses in the adjunctive antibiotic domain), the probability of reaching a non-inferiority conclusion in the EOS domain (analogous to type I error) within the maximum sample size was 0.03 for PSSA, 0.07 for MSSA and 0.04 for MRSA. When all odds ratios were set to a 0.75 in all domains (a ‘moderate’ effect size), the probability of reaching a non-inferiority conclusion was 0.78 for PSSA, 0.95 for MSSA, and 0.84 for MRSA for the adult population. The silos are analysed separately, however, Bayesian hierarchical borrowing will take place, increasing power and learning across silos.

## Trial Oversight/Governance

The EOS domain is managed predominantly by the Early Oral Switch Domain-Specific Working Group (EOS DSWG), which is responsible for protocol creation, development of interventions within the domain, and assessment of relevant emerging evidence. This group reports to the Global Trial Steering Committee. In addition, the Statistical Subcommittee, a group of blinded statisticians and clinicians, are responsible for oversight of the statistical modelling, trial design and analysis and a Data and Safety Monitoring Committee oversees the SNAP trial in its entirety.
